# Acquisition learning is stronger for aversive than appetitive events

**DOI:** 10.1038/s42003-022-03234-x

**Published:** 2022-04-04

**Authors:** Marieke E. van der Schaaf, Katharina Schmidt, Jaspreet Kaur, Matthias Gamer, Katja Wiech, Katarina Forkmann, Ulrike Bingel

**Affiliations:** 1 Radboud University Medical Centre, Department of Psychiatry, 6525 GA Nijmegen, The Netherlands; 2 Radboud University, Donders Institute for Brain Behaviour and Cognition, Centre for Cognitive Neuroimaging, Nijmegen, The Netherlands; 3 Department of Neurology, Center for Translational Neuro- and Behavioural Sciences, University Medicine Essen, Essen, Germany; 4 Department of Psychology, University of Würzburg, Würzburg, Germany; 5 Wellcome Centre for Integrative Neuroimaging (WIN), Nuffield Department of Clinical Neurosciences, University of Oxford, Oxford, OX3 9DU United Kingdom

**Keywords:** Attention, Perception, Human behaviour

## Abstract

Appetitive and aversive learning are both key building blocks of adaptive behavior, yet knowledge regarding their differences is sparse. Using a capsaicin heat pain model in 36 healthy participants, this study directly compared the acquisition and extinction of conditioned stimuli (CS) predicting pain exacerbation and relief. Valence ratings show stronger acquisition during aversive compared to appetitive learning, but no differences in extinction. Skin conductance responses and contingency ratings confirmed these results. Findings were unrelated to individual differences in pain sensitivity or psychological factors. Our results support the notion of an evolutionarily hardwired preponderance to acquire aversive rather than appetitive cues as is protective for acute aversive states such as pain but may contribute to the development and maintenance of clinical conditions such as chronic pain, depression or anxiety disorders.

## Abstract (web summary)

In this study, acquisition and extinction of conditioned stimuli are compared in a capsaicin heat pain paradigm in healthy human participants. The results demonstrate a preponderance to acquire aversive rather than appetitive cues, which could initially have a protective function but eventually contribute to the maintenance of chronic pain.

## Introduction

Successful navigation of our physical and psychological environment requires various skills—but arguably, learning from mistakes is amongst the most critical. If an encounter has led to pain in the past, animals and humans alike go to great lengths to avoid similar incidents in the future by learning to predict them from cues that signal upcoming pain. Experimental learning studies have extensively investigated this associative learning. Previously neutral stimuli of events that are repeatedly paired with unpleasant stimuli or pain (unconditioned stimulus, US) begin to function as predictive cues (conditioned stimulus, CS) and themselves become capable of triggering physiological and emotional responses to pain. However, over time the predictive value of the cue can change, and the cue might no longer be followed by pain. In this case, our representation of the association between cue and pain needs to be updated to represent the change in contingency. Although this extinction learning has been investigated in numerous studies, the principles guiding it are still subject to debate^1^. While early concepts assumed that extinction only requires erasure of previous learning, more recent accounts propose that it includes new learning that inhibits the originally formed associative learning (e.g., Pearce-Hall modell^2^).

Potential extinction and acquisition differences between appetitive and aversive, e.g., pain-related, CS–US associations might also be due to differences in their biological relevance. Although appetitive stimuli elicit approach behavior, aversive stimuli or events signal potential threats and trigger protective and avoidance behavior. Thus, a more conservative updating rule for aversive than for appetitive CS–US associations reduces the risk of underestimating the threat signaled by cues that once predicted harm, even when the chances of aversive consequences have become very small^1^. This behavior could be seen as a “better-safe-than-sorry” strategy^3^ that ensures that updating during extinction learning is delayed until a more conservative threshold for safe extinction has been reached. Overly fast acquisition and slow or incomplete extinction of aversive CS–US associations are assumed to contribute to the development and maintenance of several diseases including chronic pain^4–6^, although empirical evidence for this assumption has been inconsistent, so far^7,8^.

In order to test whether aversive and appetitive learning of predictive cues are guided by different learning rules, the two types of learning have to be compared directly within the same model and during both learning phases (i.e, acquisition and extinction). Experimentally induced tonic pain is an ideal model for this purpose as it allows to combine cues (CS) signaling aversive events (i.e., transient increases in pain) and appetitive events (i.e., transient relief from pain) within the same paradigm. To date, only a few studies have directly compared conditioned responses to cues predicting pain increase and pain relief, but their design either only included the acquisition phase^9^ or compared pain and reward in different sensory modalities (i.e., pain vs. food)^10^.

Here, we directly compared associative learning about pain and relief during both acquisition and extinction within the same sensory modality using a novel capsaicin-induced tonic heat pain model in healthy volunteers. Capsaicin increases the sensitivity to noxious and innocuous stimuli which means that safe, low-level heat stimuli can be used to induce lasting individually calibrated heat pain that resembles clinical pain but is easily modifiable through temperature manipulations^10–12^.

**Fig. 1:**
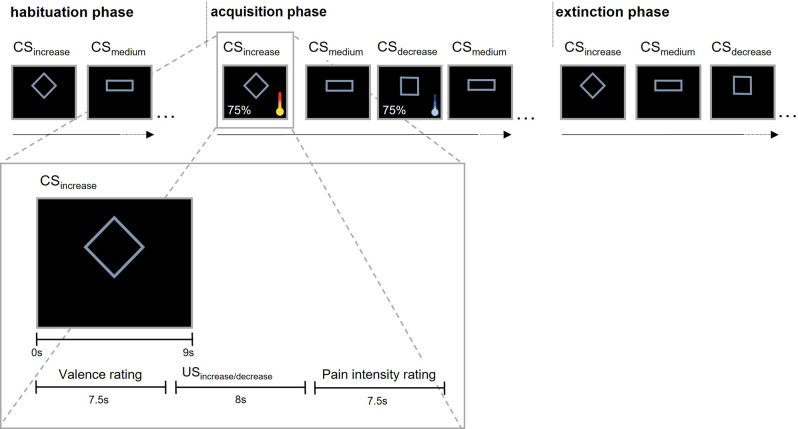
Differential conditioning paradigm. A pain intensity of about VAS 40 was induced by applying thermal heat stimuli to capsaicin-pretreated skin (tonic pain). An increase and decrease in temperature level led to pain exacerbation (VAS 80) and pain relief (VAS 0), respectively. The design consisted of habituation, acquisition, and extinction training. Assignment of geometric figures to experimental conditions was pseudo-randomized (example shown here).

Tonic, moderate pain was induced for ~45 minutes in healthy volunteers by applying thermal stimulation to the forearm that had been pretreated with capsaicin. Temperatures were individually calibrated to induce moderate ongoing pain as well as phasic increases and decreases of pain. Geometrical figures were used as visual cues. During the acquisition phase, one cue (CS_increase_) predicted pain exacerbation (US_increase_), another cue (CS_decrease_) predicted pain decrease (US_decrease_), and a third cue (CS_medium_) predicted no change in ongoing pain (US_medium_). During the extinction phase, all cues were presented without changes in temperature (US_medium_ only) (see Fig. 1). Note that in contrast to previous studies that focused on the effect of conditioning on US perception^13–16^, our study investigates changes in CS perception and learning in terms of valence ratings as the main outcome measure. Further, contingency ratings of CS–US-associations and skin conductance responses (SCR) were recorded. Pain intensity and (un)pleasantness ratings of the US were only collected to validate task manipulations.

Based on the assumption that aversive learning requires less evidence than appetitive learning, we hypothesized that CS predicting pain exacerbation is associated with enhanced differential learning (i.e., steeper acquisition slopes) and slower differential extinction slopes in valence ratings compared to CS predicting pain relief. In addition, we explored whether pain-related associative learning depended on individual pain-related cognitions^17^, including pain anxiety, pain catastrophizing, pain sensitivity and state and trait depression and stress measures, or changes in physiological responses to CS and US.

Our results show stronger acquisition during aversive compared to appetitive learning in terms of valence ratings, but no differences in extinction. These results support the notion of an evolutionarily hardwired preponderance to acquire aversive rather than appetitive cues as is protective for acute aversive states such as pain.

## Results

### US pain intensity and (un)pleasantness ratings

**Fig. 2:**
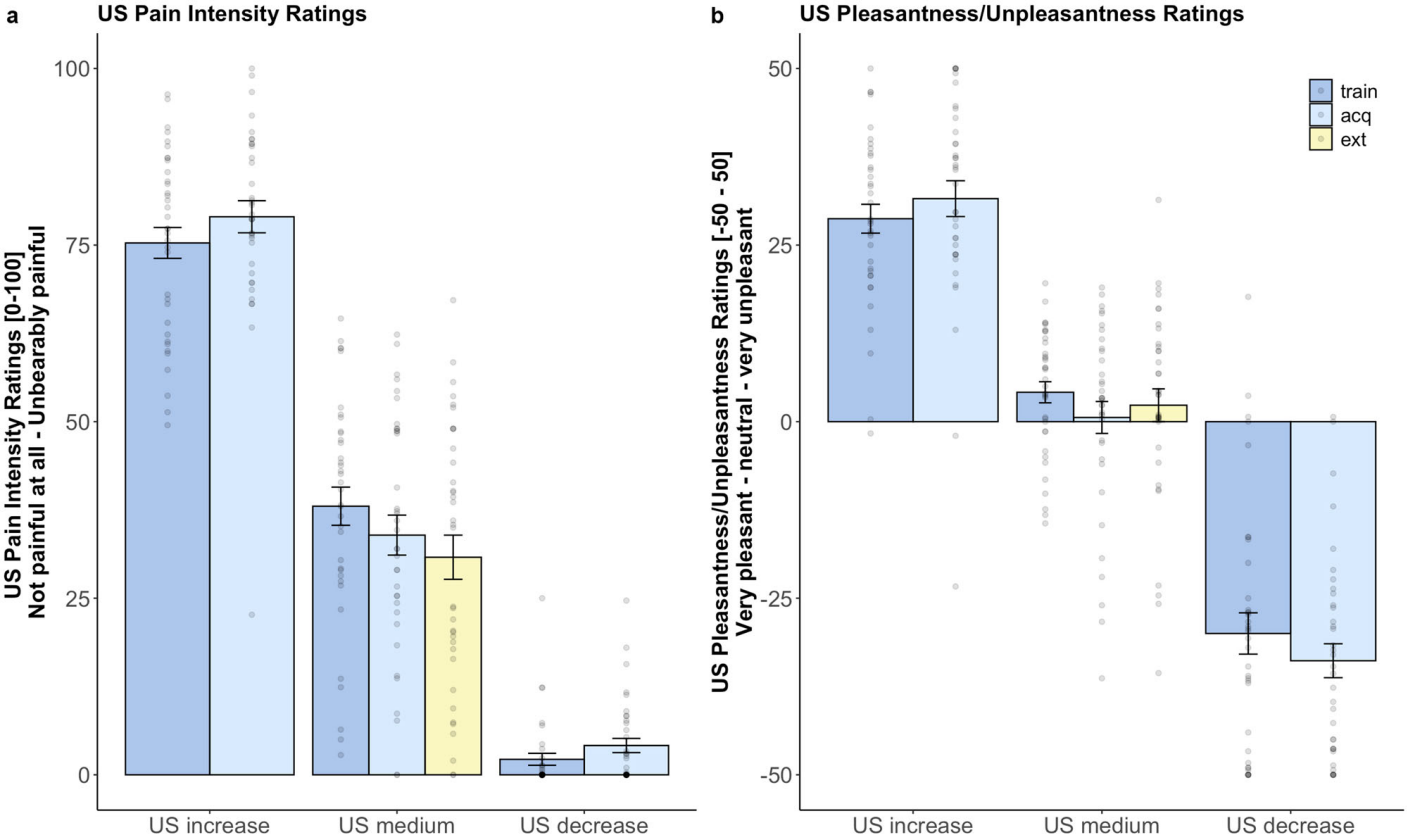
Individual pain intensity and (un)pleasantness ratings. Pain intensity (**a**) and pain (un)pleasantness (**b**) ratings during all experimental phases for all US types on 0–100 VAS (A) and −50–50 VAS (B) in mean ± standard error of the mean of raw values. Displayed are ratings for the US_increase_ and US_decrease_ during training (train) and acquisition (acq) and ratings for the US_medium_ during training, acquisition, and extinction (ext). Single data points are displayed in gray. Data are provided for *N* = 36 subjects.

Our experimental pain model successfully induced a moderate level of tonic pain (VAS 40) and the intended transient increases and decreases in pain intensity following the CS_increase_ and CS_decrease_ (see Fig. 2). Differential perception of US_increase_, US_decrease_, and US_medium_ was confirmed by analysis of pain intensity and (un)pleasantness ratings. Analyses of pain intensity ratings acquired during training and acquisition revealed a significant main effect of *US type*. As intended, the US_increase_ was rated as significantly more painful than the US_medium_ (Δ*β*: 37.27 ± 3.25; *t*(83.83) =  11.48, *p* < 0.001, *d* = 2.51) and the US_decrease_ was rated as significantly less painful than the US_medium_ (Δ*β*: −35.83 ± 3.01; *t*(99.13) = −11.89, *p* < 0.001, *d* = −2.39). US_medium_ pain intensity ratings habituated slightly over time as indicated by a significant main effect for the factor *phase* with decreasing pain intensity ratings from acquisition to extinction training (*β*: −7.22 ± 3.13; *t*(175.00) = −2.31, *p* = 0.02, *d* = −0.35). Pain intensity ratings for the transient US_increase_ and US_decrease_ showed no significant change over time (all *p* > 0.05).

The three US types were also rated differently with respect to (un)pleasantness as indicated by a significant main effect for the factor *US type*. US_decrease_ was rated significantly more pleasant than US_medium_ (Δ*β*: −34.15 ± 3.34; *t*(79.38) = −10.21, *p* < 0.001, *d* = −2.29; see Fig. 2) and US_increase_ was rated significantly more unpleasant than US_medium_ (Δ*β*: 24.57 ± 2.79; *t*(120.07) = 8.81, *p* < 0.001, *d* = 1.61). Importantly, (un)pleasantness ratings did not change over time for either US type which suggests that changes in valence ratings are not due to increases or decreases in US (un)pleasantness. To formally test a potential influence of US intensity on emotional learning, changes in individual US intensity ratings over time were added as covariates of no interest when analyzing CS valence ratings. None of the tested covariates improved model fit for either pain intensity or (un)pleasantness ratings.

See Supplementary Figure 1 for the development of US ratings over the course of the experimental phases.

Calibrated temperatures, ratings, and questionnaire results can be seen in Supplementary Table 1.

### Valence ratings of the conditioned stimuli

**Fig. 3:**
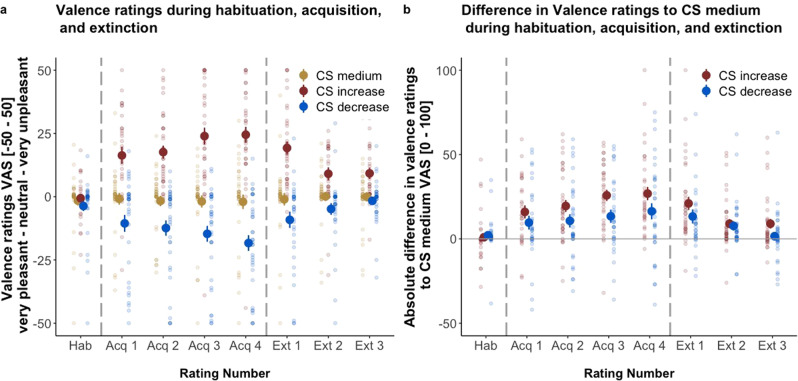
Valence ratings. **a** Valence ratings (raw value) during the habituation (Hab), acquisition (Acq 1–Acq 4), and extinction phases (Ext 1–Ext3).**b** Differential valence ratings (raw value) of CS_increase_/CS_decrease_ relative to CS_medium_ (i.e., |(CS_medium_−CS_decrease_|) during the habituation (Hab), acquisition (Acq 1−Acq 4), and extinction phases (Ext 1−Ext3). An absolute difference of 0 (solid line) indicates equal valence ratings for CS_increase_/CS_decrease_ and CS_medium_. Ratings are given as means ± standard error of the mean. Single data points in transparent colors. Dashed lines separate the phases. Data are provided for *N* = 36 subjects.

Participants showed successful differential learning, i.e., an increase in negative valence for CS_increase_ and an increase in positive valence for CS_decrease_ during acquisition training as well as successful extinction, i.e., a decrease in negative valence for CS_increase_ and a decrease in positive valence ratings for CS_decrease_ during extinction training. Individual CS-specific valence ratings and differential data in valence ratings for CS_increase_ and CS_decrease_ compared with CS_medium_ are shown in Fig. 3.

Acquisition training. Valence ratings for CS_medium_ did not significantly change over the course of the acquisition phase (*β*: −0.24 ± 0.66; *t*(295.05) = −0.37, *p* = 0.71, *d* = −0.04) (Fig. 3a). Analyses of CS-specific valence ratings of acquisition training revealed a significant *time* x *CS type* interaction. Valence ratings for CS_increase_ showed an increase in negative valence over the course of the acquisition training relative to the CS_medium_ (Δ*β*: 9.39 ± 0.91; *t*(405.97) = 10.33, *p* < 0.001, *d* = 0.67). Valence ratings of CS_decrease_ became more positive over time compared to CS_medium_ ratings (Δ*β*: −3.19 ± 0.91; *t*(405.90) = −3.53, *p* < 0.001, *d* = −0.35).

The comparison of CS_increase_ and CS_decrease_ relative to CS_medium_ (i.e., (CS_increase_−CS_medium_) and (CS_medium_−CS_decrease_)) showed larger changes in valence ratings for CS_increase_ than CS_decrease_ during acquisition training (Δ*β*: 2.91 ± 1.14; *t*(224.80) = 2.56, *p* = 0.01, *d* = 0.34). To account for potential intra- and interindividual variability in absolute pain intensity and (un)pleasantness differences, we included those ratings as covariates in our analysis (calculated as (US_increase_−US_medium_) and (US_medium_−US_decrease_)). This neither revealed any significant interactions (all *p* > 0.05) nor did it improve model fit, which suggests that our results were not driven by differences in pain perception between US_increase_ and US_medium_ vs. US_decrease_ and US_medium_. Similarly, CS valence learning was not significantly influenced by US-induced changes in electrodermal activity as the inclusion of differences in SCR amplitude (calculated as (US_increase_−US_medium_) and (US_decrease_−US_medium_)) did not improve model fit. Increased learning from US_increase_ compared to US_decrease_ was not related to any of the psychological variables (all *p* > 0.05).

Extinction training. As for the acquisition training, CS-specific valence ratings obtained during the extinction training showed a significant *time* x *CS type* interaction. CS_medium_ valence ratings did not significantly change over the course of the extinction training (*β*: 0.71 ± 0.79; *t*(304.75) = 0.90, *p* = 0.37, *d* = 0.10), while relative to CS_medium_, the valence of CS_increase_ became less negative represented in an absolut decrease in numerical rating (Δ*β*: −6.28 ± 1.13; *t*(313.88) = −5.57, *p* < 0.001, *d* = −0.63), whereas the CS_decrease_ valence ratings became significantly less positive as indicated in an absolute increase in numerical rating (Δ*β*: 4.66 ± 1.12; *t*(313.04) = 4.16, *p* < 0.001, *d* = 0.47).

The direct comparison of CS_increase_ and CS_decrease_ (relative to CS_medium_) revealed no significant differences in differential extinction learning between both CS^+^ (Δ*β*: 1.51 ± 1.42; *t*(171.14) = 1.06, *p* = 0.29, *d* = 0.16).

Although there was no difference in the pace of extinction learning between both CS^+^ (i.e., CS_increase_ and CS_decrease_), we performed an explorative analysis showing incomplete extinction for the CS_increase_ only, as indicated by the difference between the last extinction rating and the valence rating during habituation prior to acquisition training. Relative to CS_medium_, valence ratings for the CS_increase_ (Δ*β*: 1.41 ± 0.36; *t*(172.54) = 3.95, *p* < 0.001, *d* = 0.46) but not for the CS_decrease_ (Δ*β*: 0.29 ± 0.35; *t*(171.31) = 0.82, *p* = 0.41, *d* = 0.12) were significantly higher at the end of extinction training than during habituation.

None of the covariates improved model fit indicating that extinction learning was not significantly influenced by psychological traits.

### Contingency ratings

**Fig. 4:**
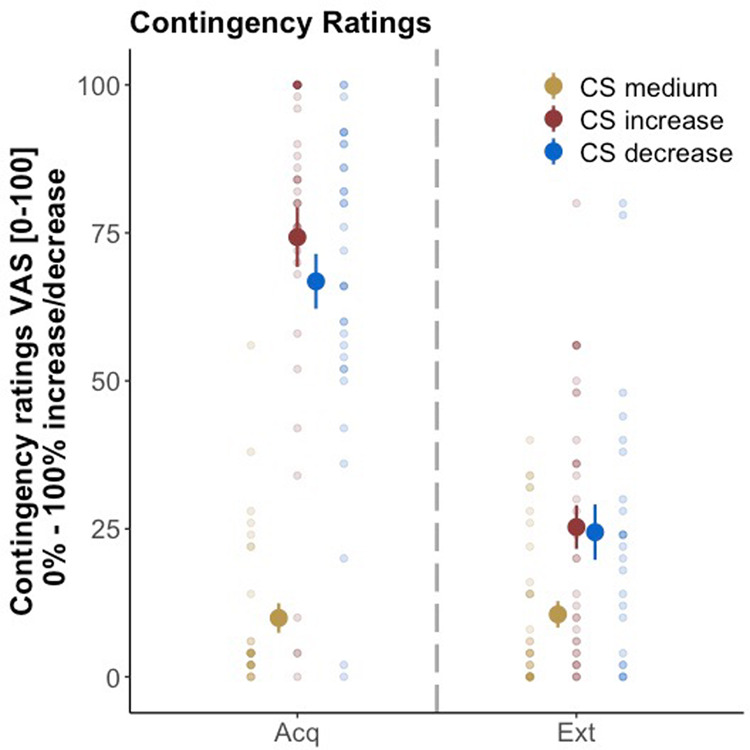
Contingency ratings. Contingency ratings (raw value) were obtained after acquisition (Acq) and extinction (Ext) training. Single data points in transparent colors. Ratings are given in means ± standard error of the mean. The dashed line separates the phases. Data are provided for *N* = 36 subjects.

Contingency ratings are displayed in Fig. 4.

Analyses revealed a significant decrease in contingency ratings from acquisition to extinction for CS_increase_ (*β*: −54.00 ± 6.09; *t*(175.00) = −8.87, *p* < 0.001, *d* = −1.34) and CS_decrease_ (*β*: −46.28 ± 6.09; *t*(175.00) = −7.60, *p* < 0.001, *d* = −1.15), but no significant change for the CS_medium_ (*β*: 5.61 ± 6.09; *t*(175.00) = 0.92, *p* = 0.36, *d* = 0.14). We also found a significant main effect of *CS type*. Across phases, both CS_increase_ and CS_decrease_ differed significantly from CS_medium_ (CS_increase_ Δ*β*: 71.06 ± 6.09; *t*(175.00) = 11.68, *p* < 0.001, *d* = 1.77; CS_decrease_ Δ*β*: 54.78 ± 6.09; *t*(175.00) = 9.00, *p* < 0.001, *d* = 1.36) and CS_increase_ yielded higher ratings than CS_decrease_ (Δ*β*: 16.28 ± 6.09; *t*(175.00) = 2.68, *p* = 0.008, *d* = 0.40). However, there was no significant interaction between the factors *CS type* and *phase* for the CS_increase_ and the CS_decrease_, indicating that changes in contingency ratings from acquisition to extinction training did not differ between both CS^+^ (Δ*β*: −7.72 ± 8.61; *t*(175.00) = −0.90, *p* = 0.37, *d* = −0.14). Including potential covariates did not improve model fit indicating that contingency awareness was not significantly influenced by psychological traits.

### Skin conductance responses

**Fig. 5:**
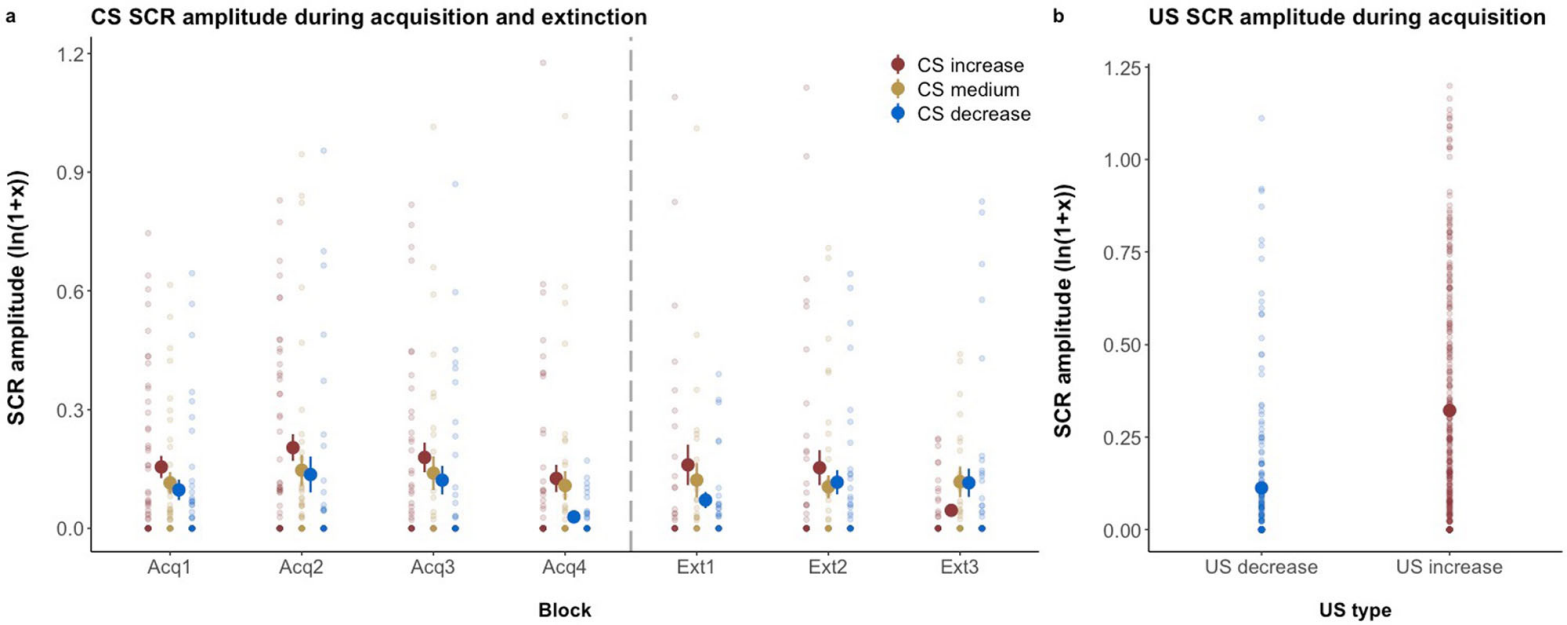
SCR amplitudes. **a** SCR amplitudes for the CS during acquisition and extinction training. **b** SCR amplitudes to the US for the entire acquisition phase. SCR is given in log-transformed means ± standard error of the mean using the natural logarithm. Single data points in transparent colors. Data provided for *n* = 31 subjects.

**Fig. 6:**
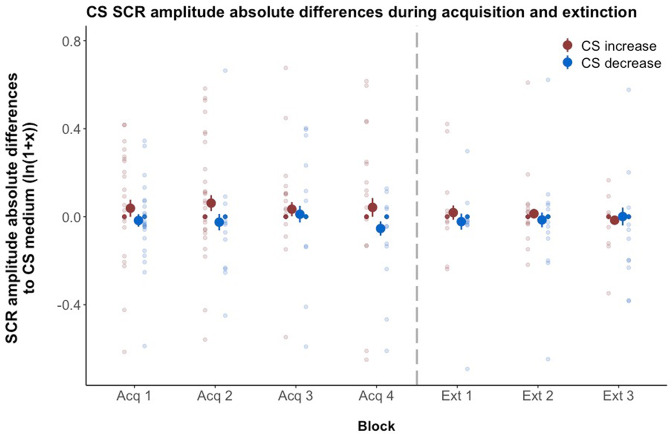
Differential SCR amplitudes. Differential SCR amplitudes for the CS during acquisition and extinction training between CS_increase_/CS_decrease_ and CS_medium_ (e.g., (CS_medium_−CS_decrease_). Ratings are given in log-transformed means ± standard error of the mean using the natural logarithm. Single data points in transparent colors. Data are provided for *n* = 30 subjects.

SCR recorded during trials without valence ratings were pooled, resulting in four pooled SCRs for acquisition training and three pooled SCRs for extinction training. The factor *time* was used as an indicator of change within each experimental phase. SCR amplitudes are shown in Figs. 5 and 6.

Unconditioned stimuli. During acquisition training, US_increase_ yielded higher SCR amplitudes than US_decrease_ (Δ*β*: 0.17 ± 0.03; *t*(30.63) = 5.51, *p* < 0.001, *d* = 1.99).

Conditioned stimuli. CS-specific SCRs collected during acquisition training showed a significant main effect of *CS type*. Consistent with results from the valence ratings, this analysis revealed overall higher SCR amplitudes for CS_increase_ than CS_decrease_ (Δ*β*: 0.06 ± 0.02; *t*(569.0) = 3.67, *p* < 0.001, *d* = 0.31). Differential effects relative to CS_medium_ (i.e., SCR CS_increase_−SCR CS_medium_ and SCR CS_decrease_−SCR CS_medium_) did not differ significantly between both CS^+^ (Δ*β*: 0.02 ± 0.02; *t*(569.21) = 1.20, *p* = 0.23, *d* = 0.10). There was no effect of *time* indicating no changes in SCR amplitudes over the course of the acquisition training for any of the *CS types* (all *p* > 0.2).

For extinction training, we found a significant effect of *CS type* with higher SCR amplitudes for CS_increase_ than CS_decrease_ (Δ*β*: 0.12 ± 0.05; *t*(380.21) = 2.70, *p* = 0.008, *d* = 0.27). Moreover, the interaction with the factor *time* indicated a stronger decrease in SCR amplitude for CS_increase_ compared to CS_decrease_ (Δ*β*: −0.05 ± 0.02; *t*(378.84) = −2.56, *p* = 0.01, *d* = −0.27) but individual SCR related to CS_decrease_ and the CS_medium_ did not significantly change over time (all *p* > 0.1). There were no significant effects when comparing differential learning of both CS^+^ (all *p* > 0.3).

## Discussion

This study used a capsaicin-heat pain model in healthy volunteers to directly compare aversive and appetitive conditioning during ongoing pain, focusing on both acquisition and extinction of CS–US associations. We showed enhanced acquisition learning during pain exacerbation compared to pain relief (i.e., steeper slopes in valence ratings for CS_increase_ versus CS_decrease_). Importantly, these findings were not explained by interindividual differences in perceived pain intensity or physiological responses (SCR) to cues signaling pain or pain relief. By contrast, no differences between extinction slopes were observed although extinction of aversive learning did not return to baseline level, while valence of relief cues was comparable to baseline values at the end of the extinction phase. These results are corroborated by contingency awareness ratings and physiological responses to the CS. Together they underscore the higher significance of pain learning during acquisition, while a different, valence-independent process may be involved in extinction learning. These results are discussed within a broader context of appetitive versus aversive learning.

Previous studies have shown successful acquisition of both appetitive and aversive CS–US associations within various domains, including pain and pain relief^9,10,18,19^, food^20^, and odors^21^. These studies were unable to draw conclusions on the relative significance of pain compared to relief learning because they either investigated only pain learning or did not directly compare pain and relief learning within the same experimental model or learning phase. Our results substantially extend these findings by directly comparing the slopes of pain and relief learning within the same domain, while controlling for physiological responses to the CS and US intensity ratings. The observation that young healthy volunteers show greater acquisition of CS_increase_ compared to CS_decrease_ is in line with our hypothesis that learning from cues that predict an increase in pain is more important than predicting pain relief. Such shifts have so far mainly been studied in other, more general models of appetitive and aversive learning such as punishment and reward learning^22–26^, and have shown to be sensitive to various manipulations including pharmacological challenges (e.g., dopamine^23,27^), stress^28–31^, and inflammation^32^, where the latter two were associated with increased aversive compared to appetitive learning.

While the acquisition was stronger when participants were presented with the CS_increase_ than the CS_decrease_, learning from the two cues did not differ during extinction training. Previous studies have so far mainly focused on the extinction of aversive CS and found evidence for a reoccurrence of previously extinguished conditioned responses in the form of spontaneous recovery, renewal, or reinstatement^1^. Although speculative at this point, this fragility of extinction could be interpreted as the result of an adaptive and evolutionarily advantageous ‘better-safe-than-sorry’ strategy as the high threshold for updating aversive CS associations should prevent the potentially costly mistake that a previously aversive predictive cue is now deemed safe when in fact it is not. However, if this was true, extinction slopes should reflect the differential pattern we see in acquisition slopes (i.e., faster extinction for CS_decrease_ than CS_increase_) which is not supported by our valence ratings showing any significant differences in extinction slopes. More recent accounts of extinction learning claim that extinction is different from acquisition, as it involves interference from previously acquired associations^1^. In line with this view, we found the biological relevance of an aversive stimulus being reflected in the stronger acquisition of negative than of positive outcomes (pain increase vs pain relief). Instead, extinction may involve a different process that is less dependent on the biological relevance of the US, at least in healthy pain-free volunteers. Of note, valence ratings for the CS_increase_ were still significantly higher than baseline ratings at the end of extinction training, indicating incomplete extinction for CS_increase_ after 12 extinction trials. In contrast, the same number of extinction trials led to complete extinction in the case of the CS_decrease_. However, the difference might also be explained by the higher negative valence of the CS_increase_ at the end of acquisition training. Even with the same extinction slope, a return to baseline would simply require more extinction trials. Hence, even though our data only indicate partial extinction for CS_increase_, these results can not be interpreted as conclusive evidence for differential extinction for CS_increase_ and CS_decrease_.

Our results showed that enhanced aversive compared to appetitive learning in healthy individuals was not linked to individual differences in trait anxiety or depression. This finding further supports our interpretation that the difference in learning might be adaptive rather than reflective of the excessive weighting of negative information as for instance seen in depression. While such a bias may constitute an evolutionary advantage in healthy individuals, especially in an acute stressful or potentially dangerous situation^33^, excessive weighting and anticipation of negative outcomes could become maladaptive and promote the development of pathological avoidance behaviors or psychopathologies like chronic pain, anxiety, and depression. Thus, chronic pain and pathological aversive states such as depression may be seen as the negative by-product of the strategy to prioritize learning from and engaging in acute aversive states.

Taken together, the current study is the first to directly compare learning and extinction of aversive and appetitive CS within the same experimental paradigm and demonstrates increased aversive versus appetitive learning that was independent of individual differences in US intensity ratings, trait anxiety, or depression. We propose that stronger learning from a cue that signaled an increase in pain (compared to one that signaled pain relief) may be reflective of a ‘better-safe-than-sorry’ strategy which ensures that aversive experiences are only no longer anticipated when it is safe to do so. Extinction, on the other hand, seems to be less dependent on the relevance of the US. The observed differences strongly indicate that extinction is more than a mirror process of acquisition but is governed by its very own learning rules.

## Methods

### Participants

In all, 43 healthy volunteers were recruited through local advertisements. Exclusion criteria were age <18 or >65 years, acute or chronic pain or other diseases including psychiatric disorders (all assessed based on self-report, clinically relevant levels of depression or anxiety (i.e., ADS-K score >18; Hautzinger et al., 2012), regular medication intake (except thyroid medication, allergy medication, occasional use of over-the-counter analgesics), body mass index >30 or <18, left-handedness, pregnancy or breastfeeding, known allergy to capsaicin and acute sunburn or other visible signs of dermatological abnormalities on the volar forearm. Only female subjects using hormonal contraceptives were included in the study. Participants were informed that the purpose of the study was to investigate visual perception and processing during noxious thermal stimulation. The study was approved by the local Ethics Committee (16-7248-BO) of the Medical Faculty, University Duisburg-Essen. All participants gave written informed consent and received monetary compensation for taking part in the study. Participants were free to withdraw from the study at any time.

Data of *n* = 7 participants were discarded from data analysis due to technical difficulties during data acquisition (*n* = 2) or perception of noxious stimuli not reaching a sufficiently painful level during calibration (*n* = 5). Behavioral data of the remaining 36 right-handed participants (19 female, age M ± SD 25.31 ± 4.29 years) were included in the analyses.

### Differential conditioning paradigm

The study used a classical conditioning paradigm with geometric figures as conditioned stimuli (CS) and contact heat as unconditioned stimuli (US). It was divided into three experimental phases (see Fig. 1); habituation (CS presentation alone), acquisition training (CS presentation with 75% reinforcement rate), and extinction training (CS presentation without reinforcement). Temperature levels for moderate pain, pain increase, and pain decrease were calibrated individually for each participant prior to the actual experiment and reassessed during a training session (see below for details).

A square, rectangle, and rhombus served as CS to predict either pain exacerbation (CS_increase_), pain relief (CS_decrease_), or no change in tonic pain of moderate-intensity (CS_medium_). Allocation of the geometric shapes to the three conditions was randomized across participants. CS was presented in blue color (RBG code: 142, 180, 227) on a black background (square: visual angle 4.99° × 4.99°, rectangle: visual angle 8.3° × 3.14°, rhombus: visual angle 7.38° × 5.36°) on a computer screen. Unconditioned stimuli consisted of a phasic increase (US_increase_), decrease (US_decrease_), or no intensity change of tonic pain (US_medium_). Tonic pain was induced by applying individually calibrated thermal stimulation (Model ATS, Pathway System, Medoc, Israel, http://www.medoc-web.com) to the site on the volar forearm that had been pretreated with capsaicin cream (1%, 8-methyl-Nvanillyl-6-nonenamide, 98%, Sigma, diluted in 5% ethanol-KY jelly)^9,18,34^. Capsaicin is the active ingredient of chili peppers that binds to vanilloid receptors (TRPV1) and increases sensitivity to thermal stimulation^9,18^. Following the application of capsaicin cream, a phasic increase and decrease of pain can be achieved by applying different levels of contact heat via a thermal stimulation device.

In all experimental phases, CS was shown on the computer screen for 9 s, followed by a black screen for 10.5 s. US were presented for 8 s, starting during the last 1.5 s of CS presentation and lasting 6.5 s into the display of a black screen. The inter-trial-interval ranged from 6 and 11 s. CS were presented in a pseudo-randomized order with no more than three consecutive presentations of the same CS and CS conditions were equally distributed within the first and second half of acquisition and extinction training. During acquisition training, the first and last CS of each type were always reinforced. For training and acquisition, six different predefined randomization protocols were used.

Presentation of visual and thermal stimuli and recording of the behavioral data were controlled using the software Presentation (www.neurobs.com).

### Behavioral outcome measures

To assess the temporal dynamics of acquisition and extinction of CS–US associations, participants were asked to provide valence ratings for each CS type throughout all experimental phases on a Visual Analog Scale, VAS (VAS: “How do you perceive this geometric figure?” -50=very pleasant, 0 = neutral, 50 = very unpleasant), which was displayed during the first 7 s of every fourth CS presentation of the same CS type (see Supplementary Material, Examples of VAS scales). To assess contingency awareness, participants were given 15 s at the end of acquisition and extinction training to rate how often each CS had been followed by a change in temperature on a VAS with anchors “100% cooling”, “no change”, and “100% heating”.

To test whether US_increase_, US_decrease,_ and US_medium_ were perceived differently, participants provided pain intensity ratings (VAS 0–100 with anchors “0 = not painful at all” and “100 = unbearably painful”) and pain (un)pleasantness ratings (VAS 0–100 with anchors “−50 = very pleasant“, “0 = neutral” and “−50 = very unpleasant”). These VAS scales were presented for 4 s after the end of every fourth US. For US_increase_ and US_decrease_, participants provided one pain intensity and (un)pleasantness rating during the training phase following calibration and three ratings during acquisition training, respectively. For US_medium_, participants rated pain intensity and (un)pleasantness once during the training phase, three times during acquisition, and five times during extinction training (see Supplementary Table 2 for details).

Prior to the experiment, participants also rated their current arousal level (“How tense do you feel at the moment?”, anchors: “not tense at all”–“extremely tense”) and pain-related fear (“How fearful are you about the upcoming pain stimulation?”, anchors: “not fearful at all”–“extremely fearful”) using a VAS. All VAS cursor positions had a random start position between 25 and 75.

### Skin conductance responses

To track changes in sympathetic arousal in response to CS and US, skin conductance was continuously recorded in all experimental phases using a BIOPAC MP150 system with the software AcqKnowledge 4.2 (BIOPAC Systems Inc). We used a bipolar recording with two disposable Ag/AgCl electrodes (0.8 cm diameter) and a conductive electrode cream (SYNAPSE®; Kustomer Kinetics). The electrodes were attached to the thenar and hypothenar eminences of the left hand. The sampling rate was set to 2 kHz and data were stored locally as text files for offline analysis.

### Study procedures

The capsaicin cream was applied to a small area of 3 × 3 cm on the volar forearm using a cotton swab and covered with a patch. After 45 minutes, the cream was removed with a dry tissue and the thermode was attached to the capsaicin-pretreated site.

### Experimental phases

#### Temperature calibration and training

In order to be able to investigate pain-related learning under realistic conditions but combined with the advantages of a controlled experimental setting, we developed an experimental model of tonic pain that would allow for repeated, deliberate variations in pain intensity. This required a calibration procedure that takes into account habituation and sensitization processes of pain that commonly occur in tonic pain models^35–37^. The following protocol is the result of extensive piloting to meet this requirement.

Temperature calibration was carried out individually for each participant to determine three temperature levels that were perceived as ‘very painful’ (VAS pain = 80), ‘moderately painful’ (VAS pain = 40), and ‘not painful’ (VAS pain = 0). To this end, a staircase procedure was applied twice, in which continuous heat was applied, starting at 28 °C and increasing in steps of 2 °C until the participant indicated a pain intensity level >70 on the VAS pain. A moderately painful temperature of VAS pain 50 was then used to determine the range for the next procedure. In this procedure, ten different temperature levels (−1.5 °C to +3.0 °C) were applied twice in a semi-randomized order. The temperature level was kept constant for 8 s before it returned to a non-painful baseline intensity of 26 °C. Using a linear regression, temperature levels corresponding to VAS60 and 80 were determined and used for tonic pain (US_medium_) and pain exacerbation (US_increase_), respectively. The temperature level for US_decrease_ was calculated as the temperature determined for tonic pain minus 15 °C (minimum 20 °C). The three temperature levels were then presented three times in a semi-randomized order to reassess pain and (un)pleasantness ratings (training session). The total time for calibration was ~20 min.

#### Habituation

After temperature calibration, contact heat at the moderate-intensity level of US_medium_ was continuously applied for the remainder of the experiment (~40 min). In addition, each CS was presented three times and participants rated their valence on a VAS (VAS: 3 ratings per CS type, nine ratings total).

#### Acquisition training

CS_increase_ and CS_decrease_ (16 CS each) were contingently paired with US_increase_ and US_decrease_, respectively (75% reinforcement rate; = 12 reinforced CS of each type, 24 reinforced CS total), while the CS_medium_ (16 CS) was not paired with changes in temperature (US_medium_). CS valence ratings were assessed on every fourth presentation of each CS (VAS, four ratings per CS type). US ratings were assessed on every third reinforced CS_increase_ and CS_decrease_ trial, resp. or every fourth CS_medium_ trial (VAS pain and VAS pleas: three ratings per US type).

#### Extinction training

The three CS types were presented without changes in temperature (12 CS each, 36 CS total) in order to extinguish the acquired CS–US associations. CS ratings were assessed on every fourth CS presentation (three ratings per CS type). US_medium_ pain intensity/or (un)pleasantness ratings were assessed five times.

Note that participants were informed about potential CS–US associations without giving further information, e.g., about different experimental phases, actual CS–US contingencies, or the absence of temperature modulation during extinction training.

### Psychological questionnaires

All participants completed the German versions of the following psychological questionnaires: (1) the Pain Anxiety Symptom Scale: PASS-D^38,39^; (2) Pain Catastrophizing Scale: PCS^40,41^; (3) Pain Sensitivity Questionnaire: PSQ-20^42^; (4) Center for Epidemiological Studies Depression Scale^43,44^; (5) State Trait Anxiety Depression Inventory: STADI^45^; and (6) Depression Anxiety Stress Scales: DASS^46^.

### Statistical analyses

The software R^47^ was used for all behavioral analyses. Linear mixed model analyses were performed on all outcome measures using the lme4 package^48^. Please see Supplementary Table 3 for details on model calculation and comparisons. All questionnaires were analyzed following their respective manual. Results with *p* < 0.05 are considered statistically significant.

Pain intensity and (un)pleasantness ratings. Analyses were performed to investigate differences in pain intensity and (un)pleasantness ratings between CS types and experimental phases. The first model included US_medium_ ratings from all experimental phases, whereas a second model included US_medium_, US_increase_, and US_decrease_ ratings from only the training phase and acquisition training. The calculated models contained the factors *phase* and *US type* (only for the model with US_increase_ and US_decrease_) as fixed effects and random intercept for the subjects to allow for subject-specific variation. The models were estimated according to the restricted maximum likelihood (REML) approach. Potential exploratory covariates were included in the models to account for their modulating influences. This comprised gender, age, pain-related fear, anxiety and depression ratings, and pain catastrophizing.

CS valence ratings. Analyses were performed to test for changes in CS valence ratings over time and differences between conditions. *Time × CS type* effects were assessed in two different analyses. The first analysis assessed the direction of learning (i.e., CS valence increased or decreased over time) and included individual CS-specific valence ratings. The second analysis assessed differential learning of cues predicting pain exacerbation and pain relief (CS_increase_ and CS_decrease_, respectively). To this end, absolute differences were calculated between the CS_increase_ and the CS_medium_ and the CS_decrease_ and the CS_mediate_ for each time point (e.g., |(CS_increase_ rating1 - CS_medium_ rating1)|)_,_ respectively. Analysis steps for both model versions were identical and are described below.

Separate models were calculated for acquisition and extinction. The last valence rating of habituation was included as a baseline rating prior to CS−US coupling in the analysis of acquisition training as the first valence rating in acquisition training was only provided after three CS−US pairings. Likewise, the last rating of acquisition training was included as a baseline in the extinction training model. To account for differences between CS types and changes over time, the factors *CS type* and *time* were included as fixed effects into the models. Random intercepts and slopes for *subjects* were included to account for subject-specific variation. The factor *time* was included as a continuous factor in order to account for increases and decreases over the course of the experimental phases. We tested whether the model fit improved when allowing variation for the factors *CS type* and *time* by adding random slopes for these factors. All models on CS valence ratings were estimated according to the REML approach. The best model was selected based on Akaike information criterion (AIC). The models including subject-specific random slopes and random effects of the factors *time* and *CS type* predicted the data best as compared to models without random slopes (acquisition training: ΔAIC = −103.1, *p* < 0.001; extinction training: ΔAIC = −123.0, *p* < 0.001, see Supplementary Methods, Supplementary Table 3 for details of model comparison).

As we found significant differences in differential pain and relief learning during acquisition training (see Results section for details), we also accounted for a potential contribution of intra-individual differences in US pain intensity and (un)pleasantness. To this end, we calculated the difference in *pain intensity* and *(un)pleasantness* ratings between US_increase_−US_medium_ and US_decrease_−US_medium_ and included those as covariates in the model. We also tested whether the US-induced SCR during acquisition training (i.e., SCR amplitude changes induced by pain increase and pain decrease) correlated with CS valence ratings by including the difference of SCR between US_increase_−US_medium_ and US_decrease_−US_medium_ in the model. Potential covariates (i.e., gender, age, pain-related fear, anxiety and depression ratings, and pain catastrophizing) were included to test for their modulatory influence.

US-CS contingency ratings. Analyses were performed to test for differences in US-CS contingency ratings between phases and CS types. The model contained the factors *phase* (i.e. acquisition and extinction) and *CS type* as fixed effects and random intercepts and slopes for *subjects*. Again, we tested whether allowing variation for the factors *CS type* and *phase* by adding random slopes for these factors, improved model fit. The model was estimated according to the REML approach. AIC was used to identify the model that fitted the data best. According to the AIC, the model with random slopes for subjects and without random effects for the factor *phase* best predicted the data (compared to random slopes excluded: ΔAIC = −6.9, *p* = 0.004). Potential covariates (see above) were included in the model to account for their modulating influences.

Skin conductance responses. Due to technical difficulties during data acquisition in *n* = 5 participants, the analysis of SCR data is based on 31 participants. The software R was used for processing and analysis of the recorded skin conductance data. First, data were down-sampled to 20 Hz and smoothed with a low pass filter with a cutoff frequency of 2 Hz. Subsequently, local minima and maxima were automatically detected in the skin conductance trace and the amplitude of stimulus-related SCR was calculated by subtracting the local minimum at the onset of the first SCR following stimulus onset from the subsequent peak. For the CS, the response window for the SCR onset was set to 1 and 4 s after CS stimulus onset^49^. For the US, the response window was set to 1–8 s after US stimulus onset, which corresponds to US duration. This larger window was chosen to account for the rise and fall time of the contact heat and the total US duration. The minimum amplitude criterion was set to 0.01 μS such that smaller responses were scored as 0 µS. These values were log-transformed using the natural logarithm to reduce the skew of the amplitude distribution^50^. In order to avoid contamination of CS and US-induced SCRs with arm and hand movement during VAS ratings, we excluded all rating trials from the SCR analysis.

Linear mixed model analyses were performed on SCR amplitudes in each experimental phase separately to test for differences between CS and US types.

Analogously to the valence analyses, we assessed SCR changes (i.e., SCR CS_increase_ or CS_decrease_ over time) and included individual CS-specific SCR data in the model. A second analysis assessed differences in SCR amplitudes between CS_increase_ and CS_decrease_ relative to the CS_medium_. To this end, absolute differences were calculated between SCR amplitudes induced by the CS_increase_ vs. CS_decrease_ (both relative to CS_medium_).

The factor *CS type* or *US type*, respectively, was included in the models as a fixed effect and random intercepts and slopes for *subjects* were included in the model. For the model investigating CS-induced SCR, the factor *time* was included as a fixed effect. To this end, SCR amplitudes between trials with valence ratings were pooled resulting in four blocks of pooled SCR amplitudes for acquisition training and three blocks for extinction training. Further, it was tested whether allowing variation for the factors *CS type*/*US type* by adding random slopes for these factors improved model fit. The models were estimated according to the REML approach. The best model fit was based on AIC.

For the analyses of CS-induced SCRs, the models without the random factor *CS type* best predicted the data as compared to the model with random slopes for CS type (acquisition training: ΔAIC = −20.80, *p* < 0.001; extinction training: ΔAIC = −5.1, *p* = 0.43). For the analysis of US-induced SCRs, the model with the random factor *US type* was not predicted due to the limited number of observations. Please see supplementary material for details in model comparisons.

### Statistics and reproducibility

Statistical analyses were conducted using the software R as described above. All information on experimental details needed on the reproducibility of the experiment is given in this manuscript and the supplementary material. Sample sizes are given in the section “Participants” and in the figure legends. Analyzed data does not include any replicates.

### Reporting summary

Further information on research design is available in the Nature Research Reporting Summary linked to this article.

## Supplementary information

Supplementary Material

Reporting Summary

## Data Availability

Behavioral and skin conductance data is provided in https://osf.io/gnk65/?view_only=dcbb22550e684a14bb3a31490ed0c6ae^51^. Further information on data will be available upon request to the corresponding author (KS). Figures 2–5 contain raw data.
