# Supplementary material for: Acquisition learning is stronger for aversive than appetitive events
Source: Commun Biol. 2022 Apr 4;5:302. doi: 10.1038/s42003-022-03234-x (PMC8979974; doi:10.1038/s42003-022-03234-x)
Supplement: Supplementary file 1 — Supplementary Material [file 42003_2022_3234_MOESM1_ESM.pdf]

Supplement to

Acquisition learning is stronger for aversive than appetitive events

Marieke E. van der Schaaf[1,2*], Katharina Schmidt[3*#], Jaspreet Kaur[3], Matthias Gamer[4], Katja Wiech[5], Katarina Forkmann[3], Ulrike Bingel[3]

*** These authors contributed equally**
# corresponding author; katharina.schmidt@uk-essen.de

[1]Radboud University Medical Centre, Department of Psychiatry, Geert Grooteplein Zuid 10, 6525 GA, Nijmegen, The Netherlands
[2]Radboud University, Donders Institute for Brain Behaviour and Cognition, Centre for Cognitive Neuroimaging, Heyendaalseweg 135, Nijmegen, The Netherlands
[3]Department of Neurology, Center for Translational Neuro- and Behavioural Sciences, University Medicine Essen, Hufelandstrasse 44, Essen, Germany
[4]Department of Psychology, University of Würzburg, Roentgenring 11, Würzburg, Germany
[5]Wellcome Centre for Integrative Neuroimaging (WIN), Nuffield Department of Clinical Neurosciences, University of Oxford, Level 6, West Wing, John Radcliffe Hospital, Oxford OX3 9DU, United Kingdom.

Supplementary Methods

Calibrated temperatures, subjective ratings and questionnaire results

Values are given in mean ± standard deviation (M ± SD) in Table 1.

Supplementary Table 1: Descriptives of study sample.

**Measurement**	**M ± SD**
Temperature $US_{increase}$ in °C	38.52 ± 5.30
Temperature $US_{decrease}$ in °C	23.32 ± 4.07
Temperature US_{medium} in °C	37.53 ± 4.12
Fear of pain rating [0-100 VAS]	15.63 ± 17.93
Arousal rating [0-100 VAS]	21.28 ± 18.62
**Questionnaires**	
ADS-K	7.49 ± 6.22
PCS	13.17 ± 8.31
PASS	26.83 ± 12.47
PSQ	28.29 ± 5.14
DASS Depression	2.13 ± 2.32
DASS Anxiety	2.58 ± 2.29
DASS Stress	4.27 ± 3.12

ADS-K: Center for Epidemiological Studies Depression Scale; PCS: Pain Catastrophizing Scale; PASS: Pain Anxiety Symptom Scale; PSQ: Pain Sensitivity Questionnaire; DASS: Depression Anxiety Stress Scales.

Supplementary Table 2: number of CS and US presentations and number of VAS-ratings

**Experimental Phase**	**# CS presentations**	**# VAS-CS**	**# US reinforcements**	**# VAS-USpain & VAS-USpleas**
**<u>Calibration:</u>**<br>Training			3x increase<br>3x decrease<br>5x medium	3x following $US_{increase}$<br>3x following $US_{decrease}$<br>5x following US_{medium}
**<u>Experiment:</u>**<br>Habituation	3x each type	3x each type		
Acquisition	16x each type	4x each type	12x pain<br>12x relief<br>24x medium	3x each type
Extinction	12x each type	3x each type	All medium	5x following US medium:<br>- 1x following $CS_{increase}$<br>- 1x following $CS_{decrease}$<br>- 3x following CS_{medium}

Supplementary Table 3: Model comparisons including and excluding random effects by AIC

**Analysis of valence ratings during the acquisition training**			
**Model number**	**Fixed effects**	**Random effects and code**	**AIC and comparison to model #x**
1	*Time × CS type*	Random intercept: *Subjects*	AIC: 4300

		Random slopes: *Subjects*<br><br>ValenceRatings ~ Time * CSType + (1 | Subjects)	
2	*Time × CS type*	Random intercept: *Subjects*<br><br>Random slopes: *Subjects, CS type*<br><br>ValenceRatings ~ Time * CSType + (1 + CSType | Subjects)	AIC: 4230.7<br>2 vs. 1: p < 0.001
3	*Time × CS type*	Random intercept: *Subjects*<br><br>Random slopes: *Subjects, Time*<br><br>ValenceRatings ~ Time * CSType + (1 + Time | Subjects)	AIC: 4303.7<br>3 vs. 1: p = 0.87<br>3 vs. 2: p < 0.001
4	*Time × CS type*	Random intercept: *Subjects*<br><br>Random slopes: *Subjects, CS type, time*<br><br>ValenceRatings ~ Time * CSType + (1 + CSType + Time | Subjects)	**AIC: 4203.1**<br>4 vs. 1: p < 0.001<br>4 vs. 2: p = 0.03<br>4 vs. 3: p < 0.001
**Analysis of valence ratings during the extinction training**			
**Model number**	**Fixed effects**	**Random effects and code**	**AIC and comparison to model #x**
1	*Time × CS type*	Random intercept: *Subjects*<br><br>Random slopes: *Subjects*<br><br>ValenceRatings ~ Time * CSType + (1 | Subjects)	AIC: 3467.4
2	*Time × CS type*	Random intercept: *Subjects*<br><br>Random slopes: *Subjects, CS type*<br><br>ValenceRatings ~ Time * CSType + (1 + CSType | Subjects)	AIC: 3375.1<br>2 vs. 1: p < 0.001
3	*Time × CS type*	Random intercept: *Subjects*<br><br>Random slopes: *Subjects, Time*<br><br>ValenceRatings ~ Time * CSType + (1 + Time | Subjects)	AIC: 3491.2<br>3 vs. 1: p = 0.97<br>3 vs. 2: p < 0.001
4	*Time × CS type*	Random intercept: *Subjects*<br><br>Random slopes: *Subjects, CS type, time*<br><br>ValenceRatings ~ Time * CSType + (1 + CSType + Time | Subjects)	**AIC: 3364.3**<br>4 vs. 1: p < 0.001<br>4 vs. 2: p = 0.03<br>4 vs. 3: p < 0.001
**Analysis of contingency ratings**			

Model number	Fixed effects	Random effects and code	AIC and comparison to model #x
1	*Phase × CS type*	Random intercept: *Subjects*<br><br>Random slopes: *Subjects*<br><br>ContingencyRatings ~ Phase * CSType + (1 | Subjects)	AIC: 2042.7
2	*Phase × CS type*	Random intercept: *Subjects*<br><br>Random slopes: *Subjects, CS type*<br><br>ContingencyRatings ~ Phase * CSType + (1 + CSType | Subjects)	AIC: 2047.5<br>2 vs. 1: p = 0.39
3	*Phase × CS type*	Random intercept: *Subjects*<br><br>Random slopes: *Subjects, Phase*<br><br>ContingencyRatings ~ Phase * CSType + (1 + Phase | Subjects)	**AIC: 2035.8**<br>3 vs. 1: p = 0.004<br>3 vs. 2: p = 0.99
4	*Phase × CS type*	Random intercept: *Subjects*<br><br>Random slopes: *Subjects, CS type, Phase*<br><br>ContingencyRatings ~ Phase * CSType + (1 + CSType + Phase | Subjects)	AIC: 2038.7<br>4 vs. 1: p = 0.008<br>4 vs. 2: p = 0.004<br>4 vs. 3: p = 0.13
**Analysis of CS SCR during acquisition training***			
**Model number**	**Fixed effects**	**Random effects and code**	**AIC and comparison to model #x**
1	*Time × CS type*	Random intercept: *Subjects*<br><br>Random slopes: *Subjects*<br><br>SCR ~ Time * CSType + (1 | Subjects)	**AIC: 238.92**
2	*Time × CS type*	Random intercept: *Subjects*<br><br>Random slopes: *Subjects, CS type*<br><br>SCR ~ Time * CSType + (1 + CSType | Subjects)	AIC: 259.72<br>2 vs. 1: p = 0.001
**Analysis of CS SCR during extinction training***			
**Model number**	**Fixed effects**	**Random effects and code**	**AIC and comparison to model #x**
1	*Time × CS type*	Random intercept: *Subjects*<br><br>Random slopes: *Subjects*<br><br>SCR ~ Time * CSType + (1 | Subjects)	**AIC: 150.74**
2	*Time × CS type*	Random intercept: *Subjects*<br><br>Random slopes: *Subjects, CS type*	AIC: 155.54<br>2 vs. 1: p = 0.43

		SCR ~ Time * CSType + (1 + CSType | Subjects)	

*Please note that model calculation with random slopes for the factor *time* was not possible due to the number of random effects exceeding the number of observations. AIC = Akaike information criterion; AIC of winning models are presented in bold font. CS: conditioned stimulus.

Supplementary results

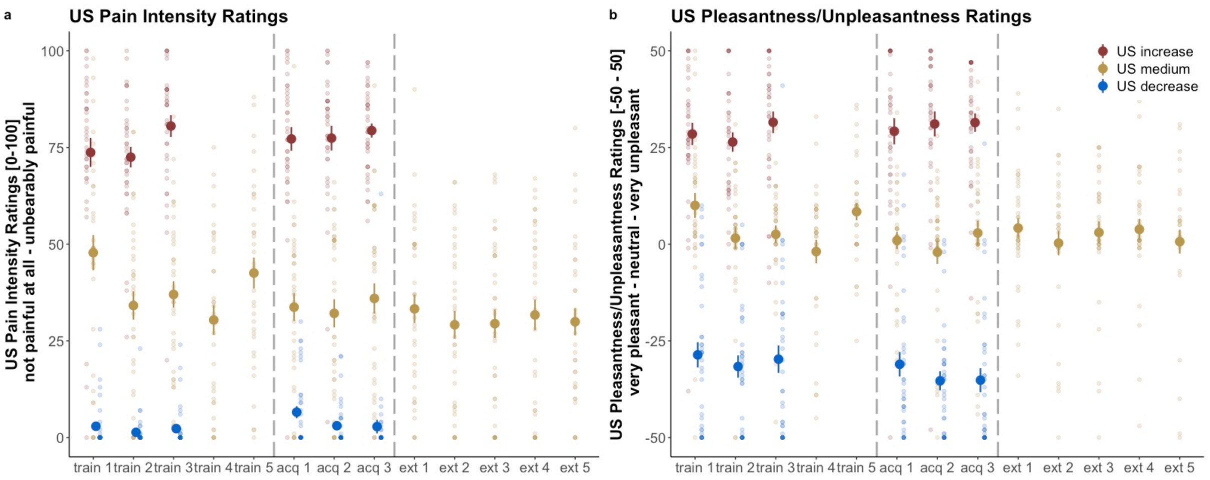

Supplementary Figure 1. Pain ratings over the course of the experiment. Pain intensity (A) and pain (un)pleasantness (B) ratings (raw values) during all experimental phases for all US types on a 0-100 VAS (A) and a -50-50 VAS (B) in mean ± SEM. Displayed are single ratings for the $US_{increase}$ and $US_{decrease}$ during training (train) and acquisition (acq) and ratings for the US_{medium} during training, acquisition, and extinction (ext).

Influence of contingency awareness on learning slopes (valence ratings)

We further included the potential covariate *contingency* into the analyses of differential learning to investigate the influence of contingency awareness on the acquisition and extinction of pain-related emotional responses, i.e., valence ratings of CS types.

For the acquisition phase, results revealed a significant interaction of the factor *time* and the covariate *contingency* for the $CS_{increase}$ (β: 0.07 ± 0.03; t(242.28) = 2.44, p = 0.02, d = 0.31) and the $CS_{decrease}$ (β: 0.08 ± 0.02; t(200.94) = 3.35, p < 0.001, d = 0.47), indicating increased differential learning in terms of valence ratings in those subjects with higher contingency awareness after acquisition for both CS+, which is in line with previous results [1]. There were no differences between CS types (Δβ: 0.01 ± 0.04; t(256.66) = 0.26, p = 0.80, d = 0.03).

Regarding the extinction phase, we observed a significant interaction of the factor *time* and the covariate *contingency* for the $CS_{increase}$ (β: -0.08 ± 0.04; t(142.23) = -2.11, p = 0.04, d = -0.35) only, indicating a steeper decrease in negative valence ratings, i.e. enhanced extinction, in those participants with higher contingency ratings after extinction. This result could be influenced by the high valence ratings at the end of the acquisition phase in those subjects with high contingency awareness. Specifically, subjects who were highly aware of the CS-US contingency during the acquisition phase

also showed higher valence learning curves during acquisition and probably also during extinction, i.e., better learning leading to better extinction. Moreover, we cannot rule out that subjects intermixed the contingency rating after the extinction phase with the acquisition phase, as we did not specifically separate the phases.

Skin conductance responses – responder analyses

We performed additional analyses including only SCR responders. For that purpose, participants showing valid US-induced SCRs (i.e., amplitudes > 0.01 µS) in less than 33% of the trials were excluded from the analyses (n=4). Here, by AIC comparison, for the analyses of CS-induced SCR amplitudes, the models with subject-specific random slopes and the random factor *CS type* best predicted the data (acquisition: ΔAIC = -10, p < 0.001; extinction: ΔAIC = -7.3, p =0.81). For the model of US-induced SCR amplitudes, calculation of the model including random effects was not possible due to a limited number of observations.

Conditioned stimuli

For the responder analysis of the acquisition phase, there were no significant effects in the analysis of neither the raw data nor the differential data (all p > 0.3).

For the extinction phase, we again observed a significant main effect of the factor *CS type* indicating higher SCR amplitudes for the $CS_{increase}$ compared to the $CS_{decrease}$ (Δβ: 0.19 ± 0.07; t(281.24) = 2.82, p = 0.005, d = 0.34). Further, the factor *block* again indicated decreases of the SCR amplitude for the $CS_{increase}$ compared to the $CS_{decrease}$ (Δβ: -0.08 ± 0.03; t(279.89) = -2.76, p = 0.006, d = -0.33) but no main effect for changes over time for the $CS_{decrease}$ and the CS_{medium} (all p > 0.2). Again, there were no significant results when comparing differential learning of both CS^+ (all p > 0.3).

Unconditioned stimuli

For the responder analysis, results also revealed significantly higher SCR amplitudes for the $US_{increase}$ as compared to the $US_{decrease}$ (Δβ: 0.30 ± 0.06; t(25.27) = 5.10, p < 0.001, d = 2.07).

Examples of VAS scales provided within the experimental paradigm

Pain Intensity Rating: “How painful was this temperature stimulus? 0 = not painful at all and 100 = unbearably painful)

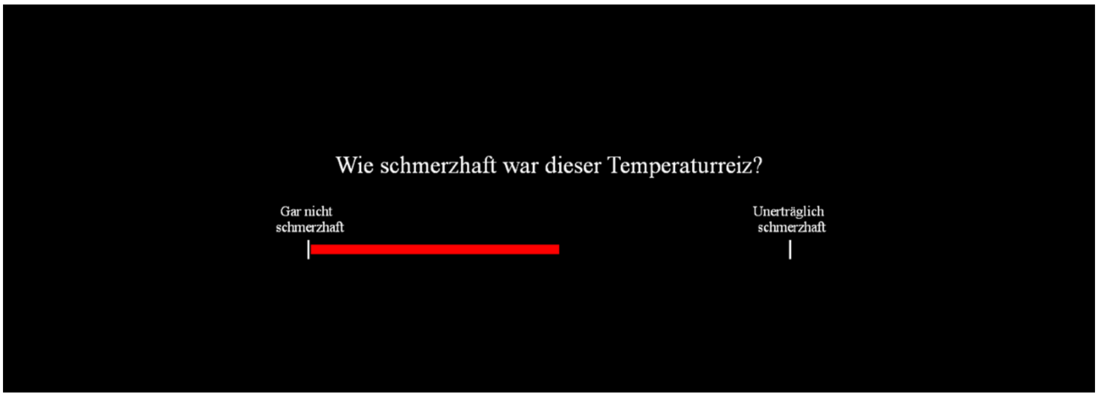

Pain (Un)Pleasantness Rating: “How pleasant/unpleasant was this temperature stimulus?” -50 = very pleasant, 0 = neutral, 50 = very unpleasant

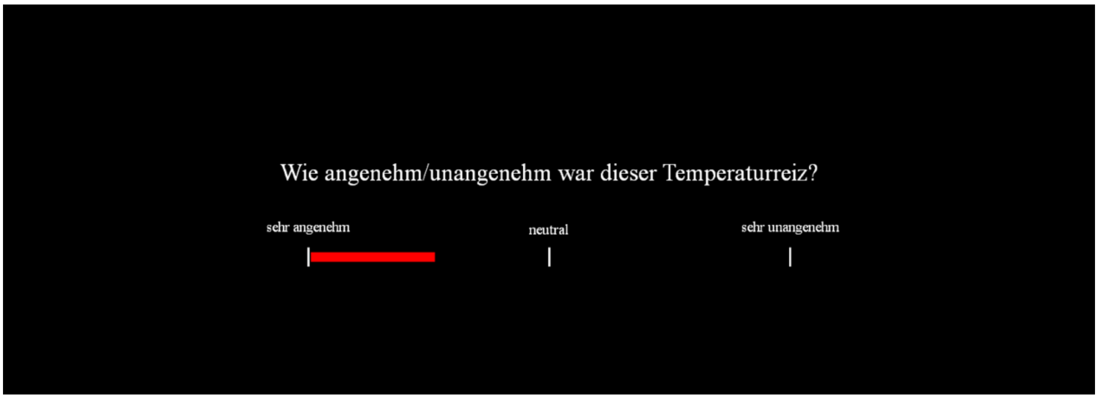

Valence Rating: “How do you perceive this geometric figure?” -50 = very pleasant, 0 = neutral, 50 = very unpleasant

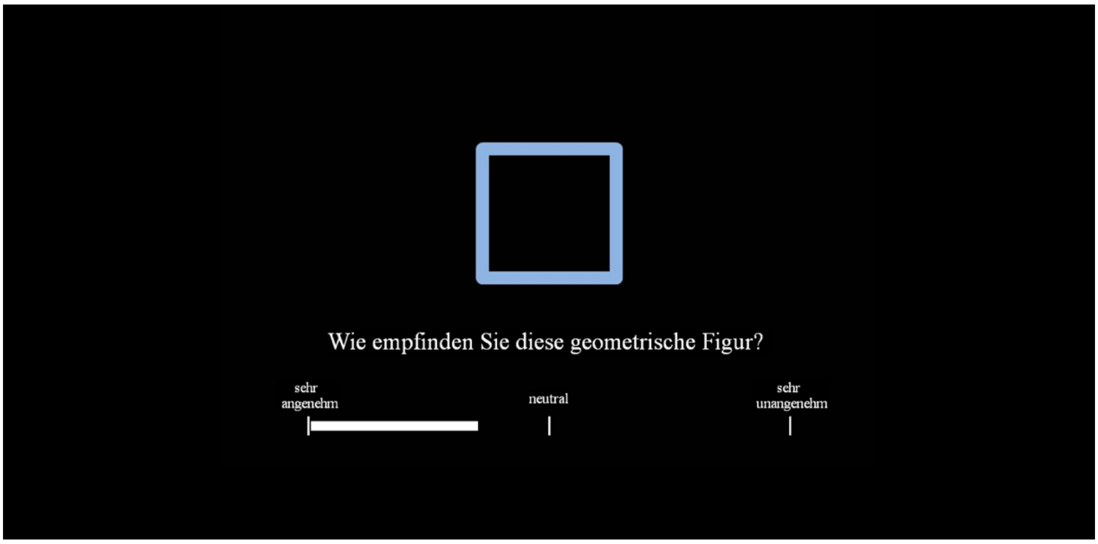

Supplementary References

1 Labrenz, F., Icenhour, A., Benson, S. & Elsenbruch, S. Contingency Awareness Shapes Acquisition and Extinction of Emotional Responses in a Conditioning Model of Pain-Related Fear. *Front Behav Neurosci* **9**, 318, doi:10.3389/fnbeh.2015.00318 (2015).
