# Supplementary material for: Acquisition learning is stronger for aversive than appetitive events
Source: Commun Biol. 2022 Apr 4;5:302. doi: 10.1038/s42003-022-03234-x (PMC8979974; doi:10.1038/s42003-022-03234-x)
Supplement: Supplementary file 2 — Reporting Summary [file 42003_2022_3234_MOESM2_ESM.pdf]

nature research

Corresponding author(s): Katharina Schmidt
Last updated by author(s): Jan 28, 2022

Reporting Summary

Nature Research wishes to improve the reproducibility of the work that we publish. This form provides structure for consistency and transparency in reporting. For further information on Nature Research policies, see our Editorial Policies and the Editorial Policy Checklist.

Statistics

For all statistical analyses, confirm that the following items are present in the figure legend, table legend, main text, or Methods section.

n/a	Confirmed	
☐	☒	The exact sample size (n) for each experimental group/condition, given as a discrete number and unit of measurement
☐	☒	A statement on whether measurements were taken from distinct samples or whether the same sample was measured repeatedly
☐	☒	The statistical test(s) used AND whether they are one- or two-sided<br>*Only common tests should be described solely by name; describe more complex techniques in the Methods section.*
☐	☒	A description of all covariates tested
☐	☒	A description of any assumptions or corrections, such as tests of normality and adjustment for multiple comparisons
☐	☒	A full description of the statistical parameters including central tendency (e.g. means) or other basic estimates (e.g. regression coefficient) AND variation (e.g. standard deviation) or associated estimates of uncertainty (e.g. confidence intervals)
☐	☒	For null hypothesis testing, the test statistic (e.g. F, t, r) with confidence intervals, effect sizes, degrees of freedom and P value noted<br>*Give P values as exact values whenever suitable.*
☒	☐	For Bayesian analysis, information on the choice of priors and Markov chain Monte Carlo settings
☐	☒	For hierarchical and complex designs, identification of the appropriate level for tests and full reporting of outcomes
☐	☒	Estimates of effect sizes (e.g. Cohen's d, Pearson's r), indicating how they were calculated

Our web collection on statistics for biologists contains articles on many of the points above.

Software and code

Policy information about availability of computer code

Data collection	Neurobs Presentation and AcqKnowledge 4.2 was used to save the data.
Data analysis	R and R Studio were used to analyze the data.

For manuscripts utilizing custom algorithms or software that are central to the research but not yet described in published literature, software must be made available to editors and reviewers. We strongly encourage code deposition in a community repository (e.g. GitHub). See the Nature Research guidelines for submitting code & software for further information.

Data

Policy information about availability of data

All manuscripts must include a data availability statement. This statement should provide the following information, where applicable:
- Accession codes, unique identifiers, or web links for publicly available datasets
- A list of figures that have associated raw data
- A description of any restrictions on data availability

Data is provided under https://osf.io/gnk65/?view_only=dcbb22550e684a14bb3a31490ed0c6ae. Further information on data will be available upon request to the corresponding author (KS). Figures 2-5 contain raw data.

Field-specific reporting

Please select the one below that is the best fit for your research. If you are not sure, read the appropriate sections before making your selection.

☐ Life sciences ☒ Behavioural & social sciences ☐ Ecological, evolutionary & environmental sciences

For a reference copy of the document with all sections, see nature.com/documents/nr-reporting-summary-flat.pdf

Behavioural & social sciences study design

All studies must disclose on these points even when the disclosure is negative.

Study description	Quantitative experimental study
Research sample	36 Young healthy volunteers (19 female, age M±SD 25.31±4.29 years)
Sampling strategy	Sample size is comparable to recent experimental behavioral studies using the same or a comparable experimental design
Data collection	Data was collected via computer, Neurobs Presentation and a Biopac System (BIOPAC MP150, AcqKnowledge 4.2). Participant and researcher were present. Researcher was not blinded to experimental conditions.
Timing	Data collection took place between 03 and 06/2018.
Data exclusions	Exclusion criteria were pre-established. Data of n=7 participants were discarded from data analysis due to technical difficulties during data acquisition (n=2) or perception of noxious stimuli not reaching a sufficiently painful level during calibration (n=5).
Non-participation	No participcants dropped out or declined participation.
Randomization	No allocation to groups. Within-subject design.

Reporting for specific materials, systems and methods

We require information from authors about some types of materials, experimental systems and methods used in many studies. Here, indicate whether each material, system or method listed is relevant to your study. If you are not sure if a list item applies to your research, read the appropriate section before selecting a response.

Materials & experimental systems

n/a	Involved in the study	
☒	☐	Antibodies
☒	☐	Eukaryotic cell lines
☒	☐	Palaeontology and archaeology
☒	☐	Animals and other organisms
☐	☒	Human research participants
☒	☐	Clinical data
☒	☐	Dual use research of concern

Methods

n/a	Involved in the study	
☒	☐	ChIP-seq
☒	☐	Flow cytometry
☒	☐	MRI-based neuroimaging

Human research participants

Policy information about [studies involving human research participants]()

Population characteristics	see above
Recruitment	Local advertisement. A bias regarding the willingness to participate in a pain study cannot be ruled out.
Ethics oversight	Ethics Committee University Duisburg-Essen (16-7248-BO).

Note that full information on the approval of the study protocol must also be provided in the manuscript.
